# Leptin, IL-6, and suPAR reflect distinct inflammatory changes associated with adiposity, lipodystrophy and low muscle mass in HIV-infected patients and controls

**DOI:** 10.1186/s12979-015-0036-x

**Published:** 2015-08-04

**Authors:** Anne Langkilde, Janne Petersen, Jens Henrik Henriksen, Frank Krieger Jensen, Jan Gerstoft, Jesper Eugen-Olsen, Ove Andersen

**Affiliations:** Clinical Research Centre, Copenhagen University Hospital, Hvidovre, Kettegård Alle 30, DK-2650 Hvidovre, Denmark; Department of Clinical Physiology and Nuclear Medicine, Copenhagen University Hospital, Hvidovre, Kettegård Alle 30, DK-2650 Hvidovre, Denmark; Department of Radiology, Copenhagen University Hospital, Hvidovre, Kettegård Alle 30, DK-2650 Hvidovre, Denmark; Department of Infectious Diseases, Copenhagen University Hospital, Rigshospitalet, Blegdamsvej 9, DK-2100 København Ø, Denmark; Department of Infectious Diseases, Copenhagen University Hospital, Hvidovre, Kettegård Alle 30, DK-2650 Hvidovre, Denmark

**Keywords:** Inflammation, Aging, Sarcopenia, HIV, suPAR, Obesity, Lipodystrophy, IL-6, Leptin

## Abstract

**Background:**

HIV-infected patients could exhibit accelerated ageing, since age-associated complications like sarcopenia; increased inflammation; lipodystrophy with loss of subcutaneous adipose tissue and/or gain of visceral adipose tissue (VAT); and cardiovascular disease occur at an earlier age. Inflammation is involved in age-associated complications. However, it is not understood whether it is the same inflammatory changes that are involved in the various ageing-associated complications. Our objective was to study whether leptin, interleukin 6 (IL-6), and soluble urokinase plasminogen activator receptor (suPAR) were associated distinctively with adiposity, lipodystrophy and sarcopenia, in HIV-infected patients and healthy Controls.

**Results:**

Systemic leptin levels were significantly higher in patients with lipodystrophy than without, whereas there was no difference in IL-6 or suPAR levels. Leptin was significantly positively associated with fat mass index (FMI) and abdominal VAT, but not with lean mass index (LMI). IL-6 was significantly associated with both FMI and VAT, and low LMI. High suPAR was associated with low LMI, and weakly with high FMI and VAT.

**Conclusions:**

Leptin reflected adiposity- and lipodystrophy-related inflammation, but not sarcopenia. IL-6 reflected both adiposity-, but also sarcopenia-related inflammation; and suPAR was a marker of sarcopenia-related inflammation. Our results indicate that different inflammatory processes can be active simultaneously contributing to the systemic low grade inflammatory state. Identifying major contributors to circulating leptin, IL-6, and suPAR levels could levels could therefore improve our understanding of which inflammatory processes are involved in the various age-related complications.

## Background

Inflammation could be a driver of biological ageing, since inflammation increases with age; is elevated in ageing-associated conditions like sarcopenia, and dysmetabolism; and is a risk factor for cardiovascular disease, type 2 diabetes, cancer, and overall mortality [1–6]. Accordingly, HIV-infected patients could exhibit accelerated ageing, as combination antiretroviral therapy (cART)-treated HIV-infected patients exhibit higher inflammatory levels and develop cardiovascular disease, type 2 diabetes, and maybe sarcopenia at a younger age than population controls [7–11]. Moreover, 30–60 % of HIV-infected patients exhibit altered adipose tissue (AT) distribution termed lipodystrophy. Lipodystrophy is characterised by loss of SAT (lipoatrophy) and/or gain of visceral adipose tissue (VAT) (lipohypertrophy), resembling an extreme form of age-associated AT redistribution. Lipodystrophy is caused by a combination of the infection and cART [12–16], and is associated with insulin resistance, systemic and adipose tissue inflammation [17, 18].

It is well-established that inflammation is elevated in the majority of age-associated complications. However, it is still not understood whether this is a general inflammatory mechanism, or whether the inflammatory biomarkers reflect different co-existing pathophysiological processes. A major limitation for understanding this, has been inconsistent adjustment for confounders with major impact on inflammation, such as not assessing muscle mass when assessing fat mass. Our aim was therefore to study whether leptin, interleukin 6 (IL-6), and soluble urokinase plasminogen activator receptor (suPAR) were associated distinctively with adiposity, lipodystrophy and sarcopenia, in HIV-infected patients and healthy Controls.

Leptin is a central adipokine produced in levels proportional to fat cell mass [19, 20]. Leptin integrates inflammation, metabolism and neuroendocrine signalling thereby regulating energy consumption and storage. Obesity is associated with increased leptin levels, and leptin resistance may be a characteristic of obesity contributing to insulin resistance and lipotoxicity [20]. Leptin levels are altered in lipodystrophy, and leptin therapy has been applied to improve insulin sensitivity in HIV-associated lipoatrophy, but with diverging results [21–23]. It is not established whether leptin levels are altered in lipodystrophy merely as a result of the AT mass, or whether AT redistribution affects leptin levels.

IL-6 is a pleiotropic cytokine produced by numerous cell types such as adipocytes, myocytes, and leukocytes. Increased IL-6 levels are associated with obesity, muscle catabolism, cardiovascular disease, and mortality [1, 24–26]. Paradoxically, IL-6 is also produced by skeletal muscle during exercise where it mediates beneficial effects of exercise, independently of muscle damage [25]. IL-6 is elevated in HIV, but IL-6 levels appear not to be affected in lipodystrophy [8, 18]. How IL-6 reflects and affects AT and muscle mass in HIV-infection and lipodystrophy is not clear.

suPAR is produced when uPAR is cleaved from the cell surface, primarily from activated T cells, monocytes, neutrophils and endothelial cells. suPAR correlates with other inflammatory proteins such as TNF-α, and suPAR levels are elevated in a variety of diseases like HIV, sepsis, cardiovascular disease, and cancer. The function of suPAR is not well-established, but patients with the highest suPAR levels generally have the worst prognosis [2, 27, 28]. In contrast to many other inflammatory markers, suPAR associates weakly with AT measures, but strongly with low lean mass [2, 29, 30]. suPAR could therefore reflect a distinct type of inflammation related to sarcopenia but not AT.

We found that leptin reflected adiposity- and lipodystrophy-related inflammation, but not sarcopenia. IL-6 reflected both adiposity-, but also sarcopenia-related inflammation; and suPAR was a marker of sarcopenia-related inflammation. Our results indicate that different inflammatory processes can be active simultaneously contributing to the systemic low grade inflammatory state.

## Results

**Fig. 1:**
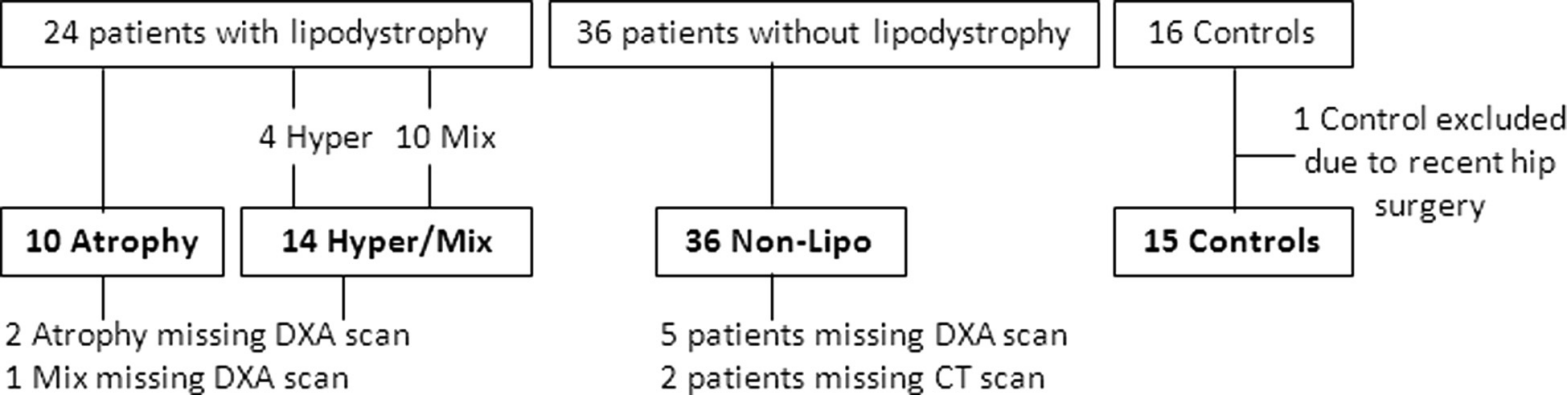
Overview of Cohort. Of the 24 HIV-infected patients with lipodystrophy, 10 had lipoatrophy (Atrophy), 4 had lipohypertrophy (Hyper), and 10 had Mixed type lipodystrophy (Mix). Patients with Hyper and Mix type lipodystrophy were grouped (Hyper/Mix). One control was excluded from data analyses due to recent hip surgery. Two patients with Atrophy, 1 Mix, and five patients without lipodystrophy (Non-Lipo) did not have DXA made, and were therefore not included in analyses of DXA scans. Two patients in the Non-Lipo did not have CT made, and were therefore not included in analyses of CT scans. Abbreviations: CT: Computed tomography; DXA: Dual energy X-ray absorptiometry

We included 60 HIV-infected patients, 24 with lipodystrophy (Lipo), 36 without lipodystrophy (Non-Lipo), and 16 healthy Controls. One Control was excluded from data analyses due to recent hip-surgery, see Fig. 1. Eight patients did not have DXA scans made. Two patients did not have CT scans made.

### Adipose tissue distribution and metabolic parameters in lipodystrophy subgroups

The Lipo group comprised: 10 patients with lipoatrophy (Atrophy), 4 patients with lipohypertrophy (Hyper), and 10 patients with both lipoatrophy and lipohypertrophy (Mix), see Fig. 1. Mean SAT was 101 cm^2^ for Atrophy, 300 cm^2^ for Hyper, and 221 cm^2^ for Mix. Mean VAT was 128 cm^2^ for Atrophy, 285 cm^2^ for Hyper, and 222 cm^2^ for Mix. The mean VAT/SAT ratio was 1.5 for Atrophy, 1.0 for Hyper, and 1.2 for Mix. Mean homeostatic model assessment of insulin resistance (HOMA-IR) was 0.9 for Atrophy; 2.1 for Hyper; and 4.9 for Mix. Metabolic syndrome was present in 10 % in Atrophy; 75 % in Hyper; and 90 % in Mix. Since lipoatrophy and lipohypertrophy could be caused by distinct disease processes, we decided to analyse Lipo in subgroups. Hyper and Mix (Hyper/Mix) were grouped for further analyses, due to the low number of patients with lipohypertrophy, and the fact that Hyper and Mix had similar VAT and metabolic risk factors compared to Atrophy.

### Baseline characteristics of atrophy, hyper/mix, non-lipo and controls

Baseline characteristics for Atrophy, Hyper/Mix, Non-Lipo and Controls are shown in Table 1. Patients in the Hyper/Mix group were significantly older than Non-Lipo. Both Atrophy and Hyper/Mix had significantly longer HIV and treatment duration, and a higher percent of patients previously treated with thymidine nucleoside reverse transcriptase inhibitors (tNRTI) than the Non-Lipo group. Smoking was significantly more prevalent, and triglyceride and very low density lipoprotein (VLDL) levels were significantly higher in Non-Lipo compared to Controls. BMI, FMI, SAT and *t*LMI was significantly lower in Atrophy compared to Non-Lipo, whereas there was no significant difference between Hyper/Mix and Non-Lipo, and Non-Lipo and Controls. In contrast, VAT was significantly higher in Hyper/Mix compared to Non-Lipo, and both Atrophy and Hyper/Mix had significantly higher VAT/SAT ratio than Non-Lipo. HOMA-IR was significantly higher and metabolic syndrome significantly more prevalent in Hyper/Mix than Non-Lipo, whereas there was no difference between Atrophy and Non-Lipo.

**Table 1:** Baseline Characteristics

Variable	Atrophy (N = 10)	Hyper/Mix (N = 4 + 10)	Non-Lipo (N = 36)	Controls (N = 15)
Demography and lifestyle				
Age (years)	50.1 (46.2; 47.5)	58.1 (54.0; 64.1)*	48.3 (40.1; 53.9)	47.6 (40.9; 62.3)
Smoking (N (%))	4 (40.0 %)	2 (14.3 %)	12 (33.3 %)	1 (6.7 %)*
Body composition				
BMI (kg/m^2^)	21.1 (19.4; 24.1)*	27.2 (24.7; 29.3)	25.5 (23.2; 28.2)	25.4 (22.2; 29.7)
FMI (kg/m^2^)	2.4 (1.6; 3.2)*	5.4 (4.0; 7.1)	4.8 (3.6; 6.7)	4.8 (4.4; 7.5)
VAT (cm^2^)	136.7 (91.7; 160.4)	251.0 (178.9; 280.9)*	136.4 (96.8; 194.3)	120.2 (93.5; 159.8)
SAT (cm^2^)	105.5 (56.4; 129.1)*	254.8 (203.2; 294.6)	197.4 (152.1; 264.1)	253.5 (146.2; 310.9)
VAT/SAT	1.4 (1.1; 1.8)*	0.8 (0.7; 1.6)*	0.7 (0.5; 0.9)	0.5 (0.4; 0.8)
*t*LMI (kg/m^2^)	17.1 (15.7; 18.4)*	20.3 (18.4; 20.6)	18.4 (17.1; 20.6)	18.4 (18.0; 20.6)
*l*LMI (kg/m^2^)	5.5 (5.0; 6.6)	6.3 (6.0; 6.7)	6.1 (5.7; 6.7)	6.5 (6.2; 7.0)
Metabolism				
Triglyceride	1.3 (1.1; 2.1)	1.4 (1.2; 2.4)	1.6 (1.0; 2.0)	0.9 (0.7; 1.1)*
Cholesterol	5.3 (4.6;6.4)	5.5 (4.8; 6.6)	5.4 (4.8; 6.4)	5.4 (4.7; 5.7)
HDL	1.4 (1.2; 1.5)	1.3 (1.0; 1.5)	1.3 (1.1; 1.5)	1.4 (1.3; 1.6)
VLDL	0.6 (0.5; 0.9)	0.5 (0.5; 0.9)	0.7 (0.5; 0.9)	0.4 (0.3; 0.5)*
LDL	3.2 (2.5; 4.2)	3.2 (2.7; 3.8)	3.4 (3.0; 4.5)	3.1 (2.6; 3.8)
HOMA-IR	0.9 (0.5; 1.2)	2.4 (1.6; 4.1)*	0.9 (0.5; 2.1)	0.5 (0.5; 1.3)*
Metabolic syndrome, N(%)	1 (10.0 %)	12 (85.7 %)*	14 (38.9 %)	2 (13.3 %)
HIV				
HIV duration (years)	19.5 (17.8; 25.6)*	20.8 (16.5; 24.4)*	11.0 (4.9; 16.2)	-
cART duration (years)	15.3 (12.6; 18.4)*	15.2 (13.6; 16.3)*	5.8 (3.5; 13.0)	-
Current tNRTI	0 (0 %)	0 (0 %)	1 (2.8 %)	-
Previous tNRTI	9 (100.0 %)*^a^	14 (100.0 %)*	18 (53.0 %)^a^	-
HIV RNA ≤ 20 copies/mL	9 (90.0 %)	14 (100.0 %)	32 (88.9 %)	-
CD4+ T cells/μL	716 (569; 830)	593 (534; 910)	535 (393; 736)	739 (598; 880)*
CD8+ T cells/μL	1090 (802; 1160)	654 (583; 829)	821 (597; 1160)	397 (281; 493)*
CD4+:CD8+ ratio	0.7 (0.6; 0.8)	1.0 (0.7;1.3)	0.7 (0.5; 0.9)	1.9 (1.5; 3.0)*

The table depicts median (25; 75 percentiles). *:*p* < 0.05 between groups and Non-Lipo. ^a^Previous treatment data missing from one patient in Atrophy and two patients in the Non-Lipo group

*Abbreviations*: *BMI* body mass index, *cART* combination antiretroviral treatment, *FMI* fat mass index, *HDL* high density lipoprotein, *HOMA-IR* homeostatic model assessment of insulin resistance, *IL-6* Interleukin 6, *LDL* low density lipoprotein, *suPAR* soluble urokinase plasminogen activator receptor, *SAT* subcutaneous adipose tissue, *tNRTI* thymidine nucleoside reverse transcriptase inhibitors, *VAT* Visceral adipose tissue, *VLDL* very low density lipoprotein

We analysed differences between groups and Non-Lipo. Data were analysed using one-way Anova. BMI, FMI, HDL, SAT, triglycerides, VAT/SAT, VLDL, HOMA, and CD4 was analysed using Kruskal-Wallis test. Smoking, metabolic syndrome, previous tNRTI, and HIV RNA ≤ 20 copies/mL were analysed using Fisher’s exact test

### Associations between leptin, IL-6, and suPAR with age, smoking, lipodystrophy, and HIV

**Fig. 2:**
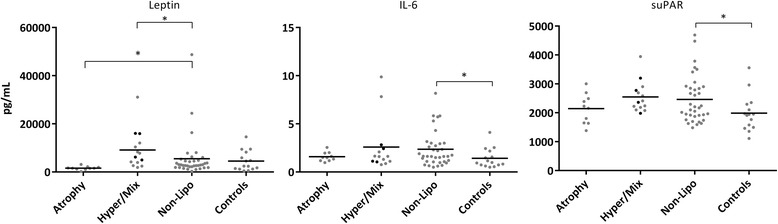
Leptin, IL-6 and suPAR levels in Atrophy, Hyper/Mix, Non-Lipo and Controls. Individuals are depicted by circles, black circles indicate individuals with lipohypertrophy. Median is presented with a line. *p < 0.05 between groups, using general linear regression

In univariate regression analyses, neither leptin nor IL-6 were associated with higher age, but suPAR tended to be (*p* = 0.05). IL-6 (*p* = 0.004) and suPAR (*p* < 0.001) were significantly higher in daily smokers than non-daily smokers, whereas leptin levels tended to be lower in daily smokers (39.5 % lower, *p* = 0.06). In univariate regression analyses, leptin levels were significantly lower in Atrophy than Non-Lipo (60 % lower, *p* = 0.007), and significantly higher in Hyper/Mix than Non-Lipo (105 % higher, *p* = 0.02), see Fig. 2 and Table 2. In contrast, there was no significant difference in IL-6 or suPAR levels between Lipo subgroups and Non-Lipo. Leptin levels were not significantly different in Non-Lipo and Controls, whereas IL-6 and suPAR were significantly higher in Non-Lipo compared to Controls (IL-6: *p* = 0.03; suPAR: *p* = 0.02), see Fig. 2 and Table 2. Furthermore, we found no associations between the biomarkers and previous treatment with tNRTI or duration of the infection or cART (data not shown).

**Table 2:** Univariate and multiple regression analyses

	Univariate % Estimate (p)			Multiple % Estimate (p)		
	Leptin	IL-6	suPAR	Leptin	IL-6	suPAR
Age (years)	0.9 (0.45)	0.5 (0.53)	0.6 (0.05)	0.01 (1.0)	0.4 (0.64)	0.5 (0.10)
Daily smoking	−39.5 (0.06)	**65.8 (0.004)**	**33.6 (<0.001)**	−32.8 (0.13)	**63.6 (0.007)**	**36.7 (<0.001)**
Atrophy vs. Non-Lipo	**−60.0 (0.007)**	−17.8 (0.41)	−11.2 (0.24)	−5.1 (0.82)	6.2 (0.81)	−5.1 (0.57)
Hyper/Mix vs. Non-Lipo	**104.5 (0.02)**	−0.1 (0.99)	6.6 (0.47)	**128.3 (<0.001)**	3.3 (0.87)	3.4 (0.63)
Non-Lipo vs. Controls	16.4 (0.59)	**58.3 (0.03)**	**23.2 (0.02)**	1.6 (0.93)	27.4 (0.21)	9.9 (0.17)
FMI (kg/m^2^)	**35.3 (<0.001)**	**8.0 (0.009)**	0.1 (0.95)	**33.5 (<0.001)**	**15.1 (<0.001)**	**3.5 (0.006)**
VAT (cm^2^)	**0.9 (<0.001)**	**0.3 (0.001)**	0.1 (0.19)	**0.8 (<0.001)**	**0.5 (<0.001)**	**0.1 (0.009)**
*t*LMI (kg/m^2^)	**26.4 (<0.001)**	−1.3 (0.70)	**−3.0 (0.03)**	0.2 (0.95)	**−10.8 (0.003)**	**−5.5 (0.002)**
*l*LMI (kg/m^2^)	**76.6 (<0.001)**	−4.9 (0.61)	**−10.6 (0.003)**	−0.6 (0.95)	**−20.7 (0.03)**	**−12.7 (<0.001)**

Biomarker levels were transformed using log_2_(x) in univariate and multiple analyses. Results are therefore back-transformed using (1-2^β^)*100 and shown as percent estimates per unit increase. Associations where *p* <0.05 are bold

*Abbreviations*: *BMI* body mass index, *FMI* fat mass index, *t*LMI total lean mass index, *l*LMI leg lean mass index, *VAT* visceral adipose tissue

Multiple analyses adjustments: Age was adjusted for group. Daily smoking was adjusted for age and group. Group analyses were adjusted for age, smoking, FMI, and *l*LMI. FMI and VAT analyses were adjusted for age, smoking, group, and *l*LMI. *t*LMI and *l*LMI analyses were adjusted for age, smoking, group, and FMI

### Univariate associations between leptin, IL-6, and suPAR with body composition

Leptin and IL-6 were strongly associated with high FMI and VAT, whereas suPAR was not (Table 2). Leptin was significantly correlated with high *t*LMI and *l*LMI; whereas suPAR was significantly correlated with low *t*LMI and *l*LMI (Table 2). IL-6 was not significantly correlated with either *t*LMI or *l*LMI.

### Interaction analyses for the univariate association between biomarkers and body composition in the four groups

**Fig. 3:**
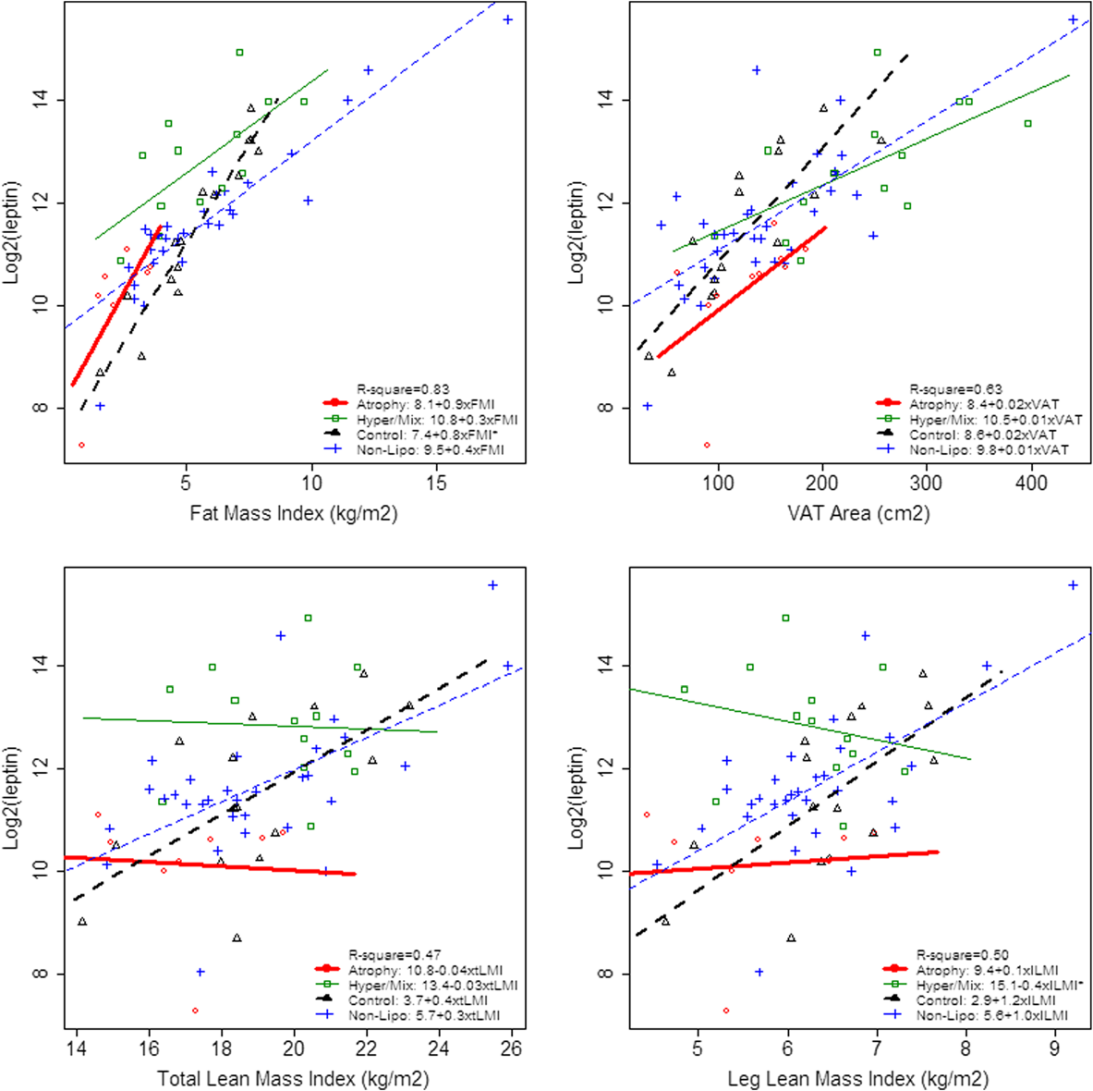
Leptin levels and Body Composition, stratified by group**.** The figure shows individuals from each group and group-specific regression lines and the equations for the regression lines. If the association is significantly different from Non-Lipo, the legend is marked with a*. Atrophy is presented by red circles and the regression line is thick, solid and red; Hyper/Mix by green and squares, and the regression line is thin, solid and green; Controls by black and triangles, and the regression line is thick, dotted and black. Non-Lipo by blue and plus, and the regression line is thin, dotted, and blue. Abbreviation: VAT: visceral adipose tissue

The association between leptin and body composition was significantly modified by group, whereas the association of IL-6 and suPAR with body composition was not, see Fig. 3. The association between leptin and FMI was stronger for Controls (*p* < 0.001) and Atrophy (*p* = 0.05) than for Non-Lipo, though only significantly different for Controls. Moreover, we found a tendency of leptin to be more strongly associated with VAT for Controls (*p* = 0.06) than for Non-Lipo. The positive association between leptin and *l*LMI was abrogated in the Hyper/Mix group (*p* = 0.01), see Fig. 3.

### Relation between fat and muscle mass

**Fig. 4:**
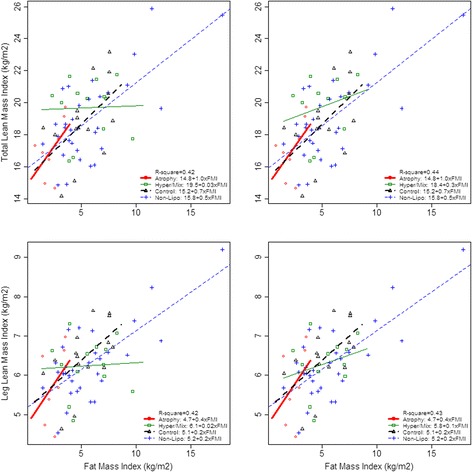
Association between fat and lean mass index, stratified by group. The figure shows individuals from each group and group-specific regression lines. Atrophy is presented by red circles and the regression line is thick, solid and red; Hyper/Mix by green and squares, and the regression line is thin, solid and green; Controls by black and triangles, and the regression line is thick, dotted and black. Non-Lipo by blue and plus, and the regression line is thin, dotted, and blue. Abbreviation: VAT: visceral adipose tissue

We investigated whether fat and muscle were associated, as found in previous studies. High FMI was significantly associated with high *t*LMI (*p* < 0.001) and *l*LMI (*p* < 0.001), see Fig. 4 left. We tested whether the association between fat and muscle mass was modified by group. In the Hyper/Mix group, FMI appeared not to be associated with *l*LMI (*p* = 0.08 versus Non-Lipo) or *t*LMI (*p* = 0.08 versus Non-Lipo). However, when removing an influential outlier from the Hyper/Mix group, the association between FMI and *l*LMI or *t*LMI was not significantly different in Hyper/Mix and Non-Lipo (*p* = 0.42; *t*LMI*: p* = 0.40 versus Non-Lipo), as seen in Fig. 4 right.

### Adjusted analyses for leptin, IL-6, and suPAR and the associations with age, smoking, lipodystrophy, and HIV

Adjusted for group, none of the biomarkers were significantly associated with age. IL-6 and suPAR were significantly elevated in daily smokers, when adjusting for age and group. We performed multiple regression analyses for the association between biomarkers, body composition and lipodystrophy, adjusted for both FMI and *l*LMI to account for the interrelation between fat and muscle mass. Results are shown in Table 2. Adjusted for age, smoking, FMI and *l*LMI we found 128.3 % higher leptin levels in Hyper/Mix compared to Non-Lipo (*p* < 0.001), but no difference between Atrophy or Controls and Non-Lipo. IL-6 and suPAR levels were not altered in Hyper/Mix or Atrophy compared to Non-Lipo, but IL-6 (*p* = 0.21) and suPAR (*p* = 0.17) were higher in Non-Lipo than Controls, though not significantly.

### Adjusted analyses for leptin, IL-6, and suPAR and the association with adipose tissue

When adjusting for age, smoking, group, and *l*LMI; leptin and IL-6 were strongly associated with FMI and VAT, whereas suPAR was only weakly associated (Table 2). The association between FMI and leptin differed between groups, and was significantly stronger for Atrophy (103.6 % per kg/m^2^, p = 0.02 vs. Non-Lipo) and Controls (72.4 % per kg/m^2^, *p* < 0.001 vs. Non-Lipo) than for Non-Lipo (30.0 % per kg/m^2^). Also the association between FMI and IL-6 (33.0 % per kg/m^2^, *p* = 0.06 vs. Non-Lipo) and suPAR (9.1 % per kg/m^2^, *p* = 0.06 vs. Non-Lipo) tended to be stronger for Controls than for Non-Lipo. We found no difference between the groups for the association between leptin, IL-6, and suPAR with VAT.

### Adjusted analyses for leptin, IL-6, and suPAR and the association with lean mass

When adjusting for age, smoking, group and FMI; IL-6 and suPAR were significantly associated with low *t*LMI and *l*LMI, whereas the positive association between leptin and lean mass was attenuated (Table 2). However, in Controls leptin was still significantly positively associated with both *t*LMI (16.2 % per kg/m^2^, *p* = 0.004 vs. Non-Lipo) and *l*LMI (59.1 % per kg/m^2^, *p* = 0.003 vs. Non-Lipo).

Exclusion of the influential outlier from the Hyper/Mix group did not change either the univariate or multiple adjusted analyses significantly. Furthermore, adjusting for HOMA-IR did not change the estimates of the multiple adjusted analyses.

### Correlation between biomarkers

We assessed the association between biomarkers, adjusted for group. Leptin and IL-6 were significantly correlated (15.5 % per pg/mL IL-6 increase; *p* = 0.02). There was no significant correlation for leptin and suPAR (-12.1 % per ng/mL suPAR increase; *p* = 0.41). IL-6 and suPAR levels correlated significantly (64.3 % per ng/mL suPAR increase; *p* < 0.001). The correlations between biomarkers did not differ significantly between groups.

## Discussion

Inflammation has been intensively studied in age-associated complications. However, it is not clear whether it is the same inflammatory changes that are central in all age-associated complications. We therefore studied leptin, IL-6 and suPAR that we hypothesised would be associated distinctively with lipodystrophy, adiposity and sarcopenia. Leptin levels were significantly elevated in lipodystrophy of the Hypertrophic or Mixed type (Hyper/Mix) compared to HIV-infected patients without lipodystrophy (Non-Lipo). In contrast, IL-6 or suPAR levels were not changed in lipodystrophy, but tended to be higher in HIV-infected patients than Controls. Leptin was a strong marker of fat mass. In contrast, suPAR showed strong associations with low lean mass, and weak associations with fat mass. IL-6 was a marker of both increased fat and low lean mass. Thus we found leptin, IL-6 and suPAR to be associated with lipodystrophy, adiposity and sarcopenia distinctively.

For the first time we show that leptin levels were significantly elevated in patients with Hyper/Mix type lipodystrophy compared to Non-Lipo, not only as a result of the fat mass or the insulin sensitivity. In contrast, leptin levels were not significantly lower in patients with lipoatrophy, than what was expected from their low fat mass. Results from other studies suggest that the elevated leptin levels do not appear to be a general feature of age-associated AT re-distribution [19]. The elevated leptin levels in Hyper/Mix type lipodystrophy could be a physiological response to counteract ectopic fat-mediated lipotoxicity, as leptin exhibits lipotoxicity-protective effects [20]. However, the higher leptin levels could also represent a state of leptin resistance contributing to insulin resistance.

IL-6 and suPAR levels were not altered in lipodystrophy of either subtype, in agreement with most studies [18, 27]. In contrast, IL-6 and suPAR levels were elevated in Non-Lipo compared to Controls, though not significantly in multiple adjusted analyses. Along with the highly elevated CD8+ T cell counts and the lower CD4+/CD8+ T cell ratio, these findings support that the immune system has not been fully normalised in HIV-infected patients despite successful treatment, in agreement with previous studies [8]. The chronic immune activation could be involved in the earlier onset of comorbidities in HIV-infected patients.

FMI and *t*LMI and *l*LMI were positively associated, in agreement with the increase in lean mass required to carry an increased fat mass previously reported [31]. However, in Hyper/Mix the association appeared disturbed, also when removing an influential outlier. Whether this is an artefact, or whether Hyper/Mix type lipodystrophy is associated with dysregulation of muscle homeostasis as in sarcopenic obesity, is not possible to determine in this study.

Leptin and IL-6 were significantly associated with FMI and VAT in both univariate and multiple regression analyses. In contrast, suPAR was only significantly associated with FMI and VAT in multiple regression analyses, in agreement with previous studies [1, 19]. This did not appear to be a result of insulin resistance, since adjusting for HOMA-IR did not change the estimates. When adjusting for fat mass, IL-6 was independently associated with low lean mass, in contrast to the univariate analysis. Moreover, we confirmed our previous finding that suPAR reflects low lean mass in HIV-infected patients and extended this to healthy Controls [29]. Neither the association with suPAR nor IL-6 and low lean mass was explained by self-reported physical activity (data not shown). The association of suPAR with low lean mass may provide new insight into why suPAR is a risk marker for mortality.

Our findings, of inflammatory biomarkers being associated distinctively with age-related processes, are in agreement with other studies, where it was reported that inflammatory biomarkers were distinctively associated with disease severity, and with risk factors leading to disease development [30, 32]. This indicates that different inflammatory processes can be active simultaneously contributing to the systemic low grade inflammatory state. CCAAT-enhancer-binding proteins (C/EBP) and specificity protein (SP)-1 are transcription factors for both the leptin and IL-6 genes. Thus, on the molecular level, the association between leptin and IL-6 and adiposity could be the result of pathways leading to C/EBP and SP-1 binding to the promoter regions of the leptin and IL-6 genes [33–35]. However, the different inflammatory processes also affect each other, as leptin and IL-6 affect the production of each other. It is not well understood, whether suPAR is regulated by, or regulates other inflammatory mediators [36, 37].

On the cellular level, adiposity-associated inflammation may result from an increased adipocyte size leading to AT stress. AT stress could induce adipocyte death, inflammation, and macrophage infiltration [38–40]. However, inflammation can also induce AT stress, thereby initiating a vicious cycle. More specifically, enlarged adipocytes may contribute significantly to the elevated leptin levels, since leptin is produced in amounts proportional to fat cell size [41]. AT-derived IL-6 can be produced by stressed adipocytes or by infiltrating macrophages. Elevated IL-6 levels may contribute to disease development, by increasing the expression of cytokines like TNF-α known to induce insulin resistance, or by inducing insulin resistance itself [5, 42, 43].

Muscle leptin expression is very small, thus leptin does not appear to be involved in muscle pathology [33]. Inflammation could cause muscle atrophy by increasing Nuclear Factor-κB expression that is known to activate atrogen gene expression in muscle [26]. IL-6 may promote muscle catabolism directly through signal transducer and activator of transcription 3 resulting in Atrogin-1 expression. However, IL-6 anabolic effects have also been reported [24]. IL-6 catabolic effects may first ensue in a persistent inflammatory state and thus be dependent on other inflammatory mediators.

The uPAR ligand urokinase plasminogen activator (uPA) exhibits muscle anabolic properties, independently of its binding to uPAR [44–47]. suPAR may directly affect muscle homeostatis by binding uPA. suPAR is cleaved from the cell surface by proteases involved in extracellular matrix degradation. The increased suPAR levels associated with low muscle mass, could therefore also be a result of the tissue re-modulation taken place during muscle atrophy [26, 28]. However, underlying pathophysiological processes could give rise to elevated suPAR and IL-6 levels, and low muscle mass, without any direct muscle atrophic effects of suPAR and IL-6.

There are some limitations to our study. The study was cross-sectional, and we could therefore not examine the role of leptin, IL-6, and suPAR in the development of complications. Moreover, our Control and lipodystrophy subgroups were small, and our findings should therefore be confirmed in larger studies. Furthermore, to understand whether the inflammatory changes identified here are indeed part of the pathophysiologic process, studies at the molecular level should be carried out. Our findings for patients with lipoatrophy are representative for relatively normo-metabolic patients, since inclusion criteria were no lipid-lowering or anti-diabetic treatment. Furthermore, our study was designed to assess lipodystrophy, and not lipodystrophy subtypes, and few patients with the lipohypertrophic phenotype were included. We therefore grouped patients with lipohypertrophy and mixed type lipodystrophy. Our findings support those from other studies, in that lipodystrophy phenotypes should be analysed separately [21, 48]*.* Finally, we could not determine the impact of the HIV infection itself, since no cART naïve HIV-infected group was included.

### Conclusion

Our findings add to the growing knowledge of inflammation involved in adiposity, adipose tissue redistribution and sarcopenia. In line with other studies, we showed that fat mass and lean mass are correlated, which could confound the relation between body composition and inflammation if both muscle and fat is not taken into account. In conclusion, we showed that leptin levels are elevated in Hyper/Mix type lipodystrophy not only as a result of the fat mass. This suggests that the leptin system is central in the pathophysiology of Hyper/Mix type lipodystrophy. In contrast, suPAR appeared to primarily reflect sarcopenia-associated inflammation, whereas IL-6 was central in both adiposity- and sarcopenia-associated inflammation. These findings suggest that leptin, IL-6 and suPAR are associated distinctively with various complications of both ageing and HIV-infection. Identifying major contributors to circulating leptin, IL-6, and suPAR levels could therefore improve our understanding of which processes leads to distinct age-related complications.

## Methods

Microdialysis results from 18 subjects of the study have been published as part of a microdialysis methodological study [49].

### Ethics

The local ethics committee of the Capital Region of Denmark (H-4-2010-045), and the Danish Data protection agency (2010-41-4952) approved the study. We carried out the study according to the declaration of Helsinki. Written informed consent was obtained from all participants.

### Subjects

We included 60 HIV-infected patients from the Department of Infectious Diseases, Copenhagen University Hospital, Hvidovre, Denmark, and 16 healthy men (Controls) from November 2010 through October 2012. Controls were recruited by advertisement at Hvidovre Hospital. Inclusion criteria were: male; age older than 18 years; White ethnicity; no current Hepatitis B (HBV) and/or C (HCV) infection; no current immunomodulating treatment or disease; no current lipid-lowering, anti-diabetic or endocrinologic treatment; no intravenous drug use. Moreover, the HIV-infected patients also had to fulfil: at least 12 month on cART; CD4+ cell counts ≥200 cells/μL; HIV-RNA <400 copies/mL. Controls were tested negative for HIV, HBV, and HCV before inclusion, and it was ensured that mean age was similar in patients and Controls.

### Study protocol

Blood samples were collected in the morning after an overnight fast of eight hours. All subjects completed a self-administered questionnaire including: tobacco use; AT distribution; and physical activity. Anthropometry was performed as in Andersen et al. [50], and included: body weight, height, waist and hip circumference. Blood pressure was measured after minimum one hour of rest. All participants were told to refrain from vigorous exercise the day before examination, physical activity on the examination day, and the use of any pain killers the week before the examination.

Whole body composition was evaluated by Dual energy X-ray absorptiometry (DXA) scan (Norland XR-36, Gammatec A/S, Værløse, Denmark). An experienced radiologist evaluated abdominal SAT and VAT mass by single slice computed tomography (CT) scan (Somatom Sensation 10, Siemens) at the upper limit of L4 as previously described in Hansen et al. [51].

Lipodystrophy was determined by physical examination by a trained examiner. Fat atrophy or hypertrophy in the face, retroauricular region, dorsocervical region, upper arms, thighs, buttocks, and abdominal subcutaneous and visceral regions was assessed. Patients were characterised as lipodystrophic (Lipo) if at least one sign of atrophy and/or hypertrophy was present, as in Andersen et al. [50]. Patients were not characterised as lipodystrophic (Non-Lipo) if no other signs of lipodystrophy than abdominal obesity were present. Patients were characterised as atrophic (Atrophy) if only signs of lipoatrophy were present; hypertrophic (Hyper) if only signs of lipohypertrophy were present, and Mixed type (Mix) if both lipoatrophy and lipohypertrophy was present.

### Body composition measures

Total and regional lean and fat mass was derived from DXA scans. Lean mass was used as a surrogate marker for muscle mass, since lean mass is primarily constituted of skeletal muscle, especially appendicular lean mass [52]. Fat and lean mass measures were constructed as previously described in Kelly et al. [53]: Fat mass index (FMI) was evaluated as: total fat mass/height^2^ (kg/m^2^). Total lean mass index (*t*LMI) as: total lean mass/height^2^ (kg/m^2^). Leg lean mass index (*l*LMI) as: lean mass of legs/height^2^ (kg/m^2^). Central SAT as: SAT area (cm^2^). Central VAT as: VAT area (cm^2^). Metabolic syndrome was assigned according to the 2009 metabolic syndrome consensus definition [54]. We used ≥94 cm as waist circumference cut-off as recommended for high risk populations.

### Blood sample measurements

CD4+ and CD8+ T-cell numbers, HIV RNA, and glucose were measured as part of the routine patient management. Insulin was measured in plasma by the Immulite® 2000 Systems (Siemens®, NY, USA). Plasma suPAR was measured with the suPARnostic® ELISA (ViroGates A/S, Birkerød, Denmark) with a mean coefficient of variation (CV) of 4 %. Plasma IL-6 was measured with Quantikine® HS-IL-6 ELISA (R&D Systems, Minneapolis, USA) with a mean CV of 8 %. Plasma leptin was measured with Quantikine® Leptin ELISA (R&D Systems, Minneapolis, USA) with a mean CV of 3 %. Insulin resistance was calculated using the homeostatic model assessment of insulin resistance (HOMA-IR) as described by Matthews et al. [55].

### Statistics

We assessed the effect of lipodystrophy on biomarker levels by comparing biomarker levels in lipodystrophy subgroups and Non-Lipo, and of HIV/cART by comparing biomarker levels in Non-Lipo and Controls. Differences in baseline characteristics between groups were analysed by one-way Anova models. If residuals were not normally distributed, we used Kruskal-Wallis tests. Group differences in smoking, HIV, previous thymidine nucleoside reverse transcriptase inhibitor (tNRTI) exposure, RNA >20 copies/mL, and metabolic syndrome were analysed by *χ*^2^ or Fisher’s exact test if less than five individuals were expected in a group.

The association of biomarkers with fat and lean mass was analysed by linear regression analyses and tested for group interactions with biomarker level as outcome. The association between fat and lean mass was analysed in a similar manner with lean mass as outcome. The association of biomarkers with age, group, smoking, fat and lean mass, was analysed in univariate analyses and multiple regression analyses adjusted for biological relevant covariates with biomarker level as outcome.

We investigated goodness of fit of the linear regression models for normal distribution of residuals and homogeneity of variance. If residuals were not normal, biomarker levels were transformed using log_2_(x) to obtain normal distributed residuals. Results from regression analyses were therefore back-transformed using 1-2^β^, and shown as percent estimates per unit increase. *P*-values less than 0.05 were considered statistically significant. The statistics program”Statistical Analysis Systems” (SAS, version 9.3; SAS Institute, Cary, NC, USA) was applied for analyses. Graphs were made using Graph Pad Prism (GraphPad Software Inc., San Diego, CA, USA) and the statistical software R (version 2.15.2, R foundation).

## Abbreviations

AT: Adipose tissue
cART: combination antiretroviral therapy
C/EBP: CCAAT-enhancer-binding proteins
CT: Computed tomography
DXA: Dual energy X-ray absorptiometry
FMI: Fat mass index
IL-6: Interleukin 6
HDL: High density lipoprotein
HOMA-IR: Homeostasis model assessment of insulin resistance
LDL: Low density lipoprotein
*l*LMI: Leg lean mass index
*t*LMI: Total lean mass index
SAT: Subcutaneous adipose tissue
Sp-1: Specificity protein 1
suPAR: Soluble urokinase plasminogen activator receptor
tNRTI: Thymidine nucleoside reverse transcriptase inhibitors
VAT: Visceral adipose tissue
VLDL: Very low density lipoprotein
